# Masking Level Differences – A Diffusion Tensor Imaging and Functional MRI Study

**DOI:** 10.1371/journal.pone.0088466

**Published:** 2014-02-18

**Authors:** David S. Wack, Paul Polak, Jon Furuyama, Robert F. Burkard

**Affiliations:** 1 Department of Nuclear Medicine, State University of New York at Buffalo, Buffalo, New York, United States of America; 2 Buffalo Neuroimaging Analysis Center, Department of Neurology, State University of New York at Buffalo, Buffalo, New York, United States of America; 3 Toshiba America Medical Systems Inc., Tustin, California, United States of America; 4 Department of Rehabilitation Science, State University of New York at Buffalo, Buffalo, New York, United States of America; University of Maryland, College Park, United States of America

## Abstract

In our previous study we investigated Masking Level Differences (MLD) using functional Magnetic Resonance Imaging (fMRI), but were unable to confirm neural correlations for the MLD within the auditory cortex and inferior colliculus. Here we have duplicated conditions from our previous study, but have included more participants and changed the study site to a new location with a newer scanner and presentation system. Additionally, Diffusion Tensor Imaging (DTI) is included to allow investigation of fiber tracts that may be involved with MLDs. Twenty participants were included and underwent audiometric testing and MRI scanning. The current study revealed regions of increased and decreased activity within the auditory cortex when comparing the combined noise and signal of the dichotic MLD stimuli (N0Sπ and NπS0) with N0S0. Furthermore, we found evidence of inferior colliculus involvement. Our DTI findings show strong correlations between DTI measures within the brainstem and signal detection threshold levels. Patterns of correlation when the signal was presented only to the right ear showed an extensive network in the left hemisphere; however, the opposite was not true for the signal presented only to the left ear. Our current study was able to confirm what we had previously hypothesized using fMRI, while extending our investigation of MLDs to include the characteristics of connecting neural pathways.

## Introduction

Masking Level Differences (MLDs) are determined with dichotic and diotic listening conditions that typically use a combination of band-pass noise and a pure tone. Interestingly, there is a substantial signal-detection advantage for the dichotic condition when either the noise or the signal is presented out-of-phase compared to the diotic condition when both the noise and signal are presented in-phase (identically) between ears. We use the notation N0S0, N0Sπ, NπS0, N0SL, and N0SR to denote whether the noise or signal are in-phase (N0 or S0), out-of-phase (Nπ or Sπ), or if the signal is presented to only one ear (SL or SR). A person's MLD can be found by subtracting the signal detection threshold for a dichotic condition (such as: N0Sπ, NπS0, N0SL, N0SR) from the signal detection threshold obtained for N0S0. The MLD for N0Sπ is usually the greatest, NπS0 is slightly less, and N0SL and N0SR are much less, with all compared to N0S0. MLDs were observed decades ago by I. Hirsch [1] and J. Licklider [2] and are still of interest for their ability to shed light on the binaural listening system.

The neural processing to enable an improved detection threshold for the dichotic MLD conditions clearly requires a combination of information from both ears. Near field studies have found neural responses to dichotic stimuli within the inferior colliculus [3], [4] and auditory cortex [5]. Auditory pathways are predominantly crossed at the level of the upper brainstem, with major crossings at the level of inferior colliculus and superior olivary nucleus. Kimura included an additional higher level crossing of verbal auditory information from the right to left hemisphere, ostensibly via the corpus callosum [6]. In this model, the processing path for verbal stimuli from the right ear would cross within the brainstem and terminate in the left hemisphere; whereas the processing path for the left ear would cross to the right hemisphere via the brainstem and ultimately cross via the corpus callosum from the right to left hemisphere. The splenium of the corpus callosum is now known to serve as a major pathway for both auditory and visual information [7]. The thalamus has been proposed to play the role of a gating system for dichotic stimuli [8], [9].

Previously we reported on fMRI activations using the dichotic listening conditions: NπS0, N0Sπ, N0SL, N0SR, Nπ, and Sπ [10]. We also used the diotic conditions N0S0, N0, and S0, and a rest or no-stimulus (NoStim) condition. fMRI is a common method to investigate functional brain activity and allows one to find regions of “activation”, which is to find locations where the T2* signal is increased in response to one condition vs. another. This allows a localization of neural sites responsible for performing a task. Using fMRI we were able to localize neural regions that required a higher (or lower) level of neural activity in subjects listening to the dichotic stimuli versus the diotic stimuli N0S0 using statistical comparisons of the corresponding T2* weighted fMRI images [10]. Activity changes typically occur in grey matter regions; however, we also detected evidence of activation within the corpus callosum. White matter activation is rare, because in these regions the image intensity is less and the actual blood flow is much lower. However, others have also reported white matter activation, especially in studies where the task required utilization of the corpus callosum [11]–[14]. We also found activation in the insulae, and in the right pulvinar thalamus that was consistent with previous findings by Hugdahl et al. [9] and surgical studies [8]. We are still confident in the involvement of the auditory cortex and inferior colliculus in the MLD, despite our previous findings not supporting this belief. Our previous study used ten subjects, and used family-wise error (FWE) corrected statistics that accounted for the analysis of the entire image volume for neural correlates of the MLD. The principles of fMRI and finding statistically significant regions of activation has been the topic of many excellent texts [15]–[17].

Other researchers have investigated dichotic listening using fMRI: Budd et al. used varying levels of interaural correlation [18]; Ernst et al. examined release from auditory masking [19]; Hall et al. investigated iterated noise and Huggins pitch using fMRI [20], [21] and Chait et al. used magnetoencephalography (MEG) [22] with a similar study; and iterated ripple noise was recently used by Barker et al. [23]. Many of these other studies were able to find neural correlates within the auditory cortex, although they used statistical metrics that utilized a-priori assumptions that there would be findings within the auditory cortex. In contrast, while we previously hypothesized neural correlates of the MLD within the auditory cortex, we took a conservative approach and used FWE correction for the entire image volume. Our approach allowed us to report on findings we did not anticipate, which we believed was appropriate as the intent of our previous study was “exploratory”.

Diffusion tensor imaging (DTI) is a technique that can assess properties of the fiber tracts. This includes the corpus callosum [24], which is known to be involved with dichotic listening [25], and auditory hallucinations [26]. Recently, DTI and fMRI were used in a dichotic listening study investigating auditory processing disorder [27]. While DTI imaging is not typically used to detect changes in processing, it can be used to spatially localize differences between populations, or to correlate with an external measure of the participants. Additionally, we wanted to focus on finding locations within in the auditory cortex. To achieve both our DTI and fMRI goals we increased the number of subjects in this study and examined specific regions where we believe that neural correlates of the MLD will be found, such as the auditory cortex. Furthermore, we hypothesize that DTI measures will correlate with signal detection performance in the regions of the corpus collosum, the superior olivary complex, lateral lemnicus, and inferior colliculus.

## Methods

The study protocol was approved by the University at Buffalo, Health Science IRB, and all participants gave their written consent prior to auditory screening. This current protocol is nearly identical to our previous study with the minor changes including the following: 20 participants were used in the present study; auditory testing was performed at the MRI scanning center immediately before scanning, with the required hearing screening threshold relaxed to 30 dB SPL; there was no minimum MLD requirement for inclusion; scanning was performed on a Toshiba Titan 3T scanner; auditory stimuli were presented using the stock auditory presentation system of the Toshiba Titan 3T scanner using insert headphones; and DTI imaging was added to the protocol.

### Auditory testing

Hearing screening was performed using tones randomly presented to the left, right, or neither ear (silence) for frequencies between 250 and 8000 Hz in octave steps. Twenty subjects with hearing thresholds better than 30 dB SPL were included for the remainder of the study. Signal detection thresholds were determined for the conditions: N0S0, N0Sπ, NπS0, N0SL, N0SR, using the same in-house software used as our previous study [10], [28]. Briefly this used a three item forced choice design, such that two presentations were just of noise and the other also contained the 500 Hz sine signal, presented in bursts lasting 250 ms with a 25 ms rise and fall time, and presented every 500 ms. Levels were tested for N0S0, N0Sπ, NπS0, N0SL, and N0SR. Each presentation lasted one second; a half second gap was used between items. The noise segments were presented at a constant level of 75 dB SPL, the signal level was first presented at a level of 80 dB SPL. The signal increased in level if the participant did not correctly choose the noise segment with the signal present, or decreased if the participant could correctly identify the noise segment containing the signal, twice in a row. Seven direction changes were used with step sizes of 8, 4, 4, 4, 2, 2, and 2 dB. Noise segments were created by randomly sampling a ∼10 minute duration of 400–600 Hz band-pass noise with 50 dB attenuation +− 100 Hz, created using an equiripple FIR filter with order 1064. From a randomly chosen noise segment, the starting sample was chosen by searching forward 2 ms to find the starting sample that maximized the correlation between the signal and noise to maintain consistency between presentations. Presentation and threshold evaluation software was developed using Matlab (The Mathworks, Natick, MA).

### fMRI conditions

Four sessions were collected, each lasting approximately 12.5 minutes. A sparse design [29], [30] was followed, such that auditory stimuli were presented for 8 seconds when there was no scanner gradient noise. Stimuli immediately preceded the scanner data collection, and followed a 1 second period of quiet. Ten conditions were used: N0S0, S0, Sπ, N0, Nπ, N0Sπ, NπS0, N0SL, N0SR, and NoStim (see Table 1). During each session each condition was presented six times, using random permutations to be balanced across the session. An added restraint was that the same condition could not end one permutation and start the next. Noise and signal combinations were constructed using the same core functions as used above for determining signal thresholds. The signal was presented within an envelope (.25 second tone burst, 50% duty-cycle) to make it distinguishable from the noise. Stimuli were presented with the narrow-band noise level of 75 dB SPL. The signal level was determined in the scanner room immediately prior to subject scanning. The level for the signal was set so the signal portion of the condition N0S0 could be barely, but reliably identified. This signal level was then used for all conditions. Participants were instructed to “listen for the signal”, prior to the start of each session. The first three volumes from each session were intentionally discarded, and were prior to the presentation of stimuli.

**Table 1:** Conditions.

Condition Name	Description
NoStim	No Stimulus presented. “Rest”.
S0	500 Hz pure tone presented in-phase to both ears.
Sπ	500 Hz pure tone presented out-of-phase to both ears.
N0	400–600 Bandpass noise presented in-phase to both ears.
Nπ	400–600 Bandpass noise presented out-of-phase to both ears.
N0S0	400–600 Bandpass noise presented in-phase to both ears,
	500 Hz pure tone presented in-phase to both ears.
N0Sπ	400–600 Bandpass noise presented in-phase to both ears,
	500 Hz pure tone presented out-of-phase to both ears.
NπS0	400–600 Bandpass noise presented out-of-phase to both ears,
	500 Hz pure tone presented in-phase to both ears.
N0SL	400–600 Bandpass noise presented in-phase to both ears,
	500 Hz pure tone presented to left ear only.
N0SR	400–600 Bandpass noise presented in-phase to both ears,
	500 Hz pure tone presented to right ear only.

The study's conditions were formed from the combination of bandpass noise, N, and a pure tone signal, S. The signal and noise could be presented in-phase between ears, i.e. a phase difference of 0, or fully out of phase between ears, i.e. π radians.

### fMRI

The sparse sequence used a 12 second repetition time (TR), a 3 second acquisition time (TA), and echo time (TE) = 40 ms. 23 slices 3 mm thick were acquired with no gap, and field of view (FOV) = 24.0 cm, using a 128×80 acquisition matrix, for an in-plane resolution of 1.9 mm×1.9 mm. This gave a nine second period that was free of the sound of the gradients. The stimulus was 8 seconds in length, was presented after 1 second of silence, and immediately preceded the scanner's acquisition. Care was taken to ensure that the inferior colliculus was included in the imaged volume. Following methodology of SPMd [31] and our previous study [10], [28], outlier voxels were identified as greater than two standard deviations from the modeled fit for each session of all participants. Individual volumes were also examined for motion from the preceding volume. Volumes having excessive motion (greater than 1 mm) or a greater than 30 fold increase in the median number of outlier voxels were eliminated from the subsequent statistical analysis. Realignment and normalization [32] of the T1 image was performed using the iterative segmentation algorithms of SPM8 (http://www.fil.ion.ucl.ac.uk/spm). The T1sequence parameters were TE/TR = 3.2/7.1 ms, inversion time (TI) = 900 ms, and flip angle = 8°. 168 slices 1 mm thick were acquired sagittally with no gap, FOV = 25.6 cm, and acquisition matrix of 256×256 for an in-plane resolution of 1 mm×1 mm. The realignment transforms together with mutual information routines of SPM were used to normalize the functional and DTI data to the MNI template. Image data were subsampled at .8×.8×.8 mm^3^, and smoothed with a 8×8×8 mm^3^ Gaussian smoothing kernel. First level statistical analysis was performed for each contrast. A second level analysis was then performed using the SPM contrast images from each participant created during the first level analysis. While determining SPM activations, we plotted the individual participant's fits to see if a participant's value was appreciably different from the others. SPM contrasts related to the MLDs were determined for N0SL vs. N0S0, N0SR vs. N0S0, N0Sπ vs. N0S0, NπS0 vs. N0S0, and NπS0 vs. N0Sπ. We used the relatively lenient threshold of p<.01, uncorrected for multiple comparisons, for the selection of regions to report. We provide both SPM t and Z scores so that the reader can consider the relative strength of each finding, and use an * to mark regions that are significant accounting for multiple comparisons within specified regions of the auditory cortex, pulvinar thalamus, and inferior colliculus, i.e. Small Volume Correction (SVC). Our small volume mask for the auditory cortex was based on the work of Rivier and Clarke [33]. Small volume masks for the inferior colliculi and pulvinar thalamus were based on a standard atlas [34]. Additional contrasts were formed between N0SL, N0SR, N0, S0, N0S0, N0Sπ, and NπS0 and NoStim.

### DTI

DTI is an MRI technique that can be used to quantify water diffusion in tissue. A diffusion image indicates preferential movement of water in a particular direction; and in the processing of these images, the three orthogonal directions, or eigenvectors, are determined and ranked by their diffusion magnitude. The mean diffusivity (MD) is the sum of diffusion in these directions. The diffusion in the primary direction is denoted as the axial diffusivity (AD), and fractional anisotropy (FA) is a relative measure of diffusion directionality and ranges from 0 (isotropic) to 1 (anisotropic). Healthy white matter tissue exhibits anisotropic diffusion, and we would expect to find higher FA and AD values in these areas. DTI was acquired with full brain coverage (30 directions with b = 1000, and one b = 0 image). Echo/repetition time for the DTI sequence were TE/TR = 82.0/9000 ms, 1 average. 46 slices 3 mm thick were acquired with no gap and FOV = 23.0 cm, using a 160×160 acquisition matrix, for an in-plane resolution of 1.4 mm×1.4 mm. Dicom images were converted to NifTI format using dcm2nii (http://www.mccauslandcenter.sc.edu/mricro/mricron/dcm2nii.html), and were corrected for eddy currents. FSL tools were used to create FA, MD, and AD image measures [35] of the DTI tensors, using FMRIB's Diffusion Toolbox – FDT v2.0 (fsl.fmrib.ox.ac.uk/fsl/fsl-4.1.9/fdt/fdt_dtifit.html). SPM was used to correlate individual signal thresholds for N0S0, N0Sπ, NπS0, N0SL, and N0SR. Regions of interest were defined as lateral lemniscus/superior olivary complex region, inferior colliculus region, and corpus callosum.

## Results

### Subjects

Ten females with mean age 26.9 (5.3) years and ten males with mean age 28.3 (8.2) years were enrolled, and all completed full testing. All were self-reported as right-handed.

### Perceptual MLDs

Mean signal thresholds for N0S0, N0Sπ, NπS0, N0SL, and N0SR were 68.9 (3.0), 52.9 (5.1), 56.0 (3.8), 61.8 (3.2), and 62.5 (3.6) dB SPL, respectively. The MLDs for N0Sπ, NπS0, N0SL, and N0SR were 16.0, 12.9, 7.1, and 6.4 dB. All participants but one, who had an MLD of 4 dB, had an MLD for N0Sπ that was greater than or equal to 10 dB. Participants' thresholds are provided in Table 2. The mean number of scan volumes eliminated from the analysis for being an outlier, as described above, averaged 2.9 scans per participant. For eleven participants, no outliers were found; for one participant, 31 outliers were found. For comparison, in our previous study, an average of 16.1 scan volumes per participant were identified as being an outlier [28]. Overall, there were more outliers identified for later runs in a scan session than for early runs.

**Table 2:** MLD.

N0S0	N0Sπ	NπS0	N0SL	N0SR	N0Sπ - N0S0	NπS0 - N0S0	N0SL - N0S0	N0SR -N0S0
69	55	55	63	63	14	14	6	6
72	52	56	64	62	20	16	8	10
73	55	63	65	69	18	10	8	4
67	39	51	61	61	28	16	6	6
67	51	55	61	61	16	12	6	6
65	54	57	60	58	11	8	5	7
70	56	58	64	66	14	12	6	4
68	52	52	62	64	16	16	6	4
72	52	52	58	60	20	20	14	12
66	54	54	62	68	12	12	4	−2
68	50	54	60	60	18	14	8	8
70	50	54	60	60	20	16	10	10
70	60	60	64	66	10	10	6	4
70	48	52	62	60	22	18	8	10
68	52	56	60	58	16	12	8	10
66	54	54	62	60	12	12	4	6
76	52	60	64	64	24	16	12	12
64	52	56	54	60	12	8	10	4
66	54	54	60	60	12	12	4	6
70	66	66	70	70	4	4	0	0

Signal detection threshold levels for noise and signal conditions for all subjects measured in dB SPL (Columns 1–5). Individual MLD levels (Columns 6–9), measured in dB.

### fMRI/DTI Contrasts

Results for the fMRI contrasts for the hypothesized regions are presented in Table 3. DTI results are presented in Table 4. For this table and the discussion relating DTI measures with signal detection thresholds, we refer to negative and positive correlations. As these tables are quite large, we refer to line numbers that are provided in these tables to guide the reader. A negative correlation correlates decreasing (improving) signal detection thresholds with an increasing DTI measure (FA, MD, or AD). A positive correlation indicates regions where an increase in a DTI measure correlates with an increase (worse performance) for the signal detection threshold.

**Table 3:** fMRI Contrasts.

Contrast	Row #	Location	MNI Max	SPM T	SPM Z	Size
N0Sπ – N0S0	1	L. Aud.	−50 −27 11	3.9*	3.3	637
	2	R. Aud.	47 −30 2	3.3	2.9	87
	3	Splenium	−1 −30 13	2.8	2.5	51
	4	CC	0 6 22	2.9	2.2	165
N0S0 – N0Sπ	5	L. Aud.	−60 −5 0	6.6*	4.7	3101
	6	L. Aud.	−32 −33 15	3.6	3.1	341
	7	CC	12 −5 34	3.4	3.0	425
NπS0 – N0S0	8	L. Aud.	−49 −26 9	3.4	3.0	850
	9	R. Aud.	64 −34 9	3.0	2.7	186
	10	L. P. Thal.	−27 −32 8	3.0	2.7	46
N0S0 - NπS0	11	L. Aud.	−56 −7 2	4.4*	3.6	1250
	12	L. Aud.	−38 −37 14	4.2*	3.5	3030
	13	R. Aud.	58 −5 −4	3.0	2.7	481
	14	R. Aud.	34 −30 14	3.1	3.8	213
	15	Med. Dorsal Thal.	7 −19 −1	3.4	3.0	952
N0SL- N0S0	16	L. Aud.	−48 −39 4	2.8	2.5	57
	17	L. P.Thal.	−18 −29 4	2.7	2.4	33
	18	L. Med. Geniculate Nucl.	−19 −13 −11	3.1	2.7	90
N0S0 - N0SL	19	L. Aud.	−38 −40 13	4.4*	3.6	1172
	20	L. Aud.	−61 −25 12	3.2	2.8	261
	21	Splenium	−5 −30 32	3.3	2.9	702
N0SR - N0S0	22	L. Aud.	−50 −45 8	3.1	2.8	584
	23	L. Aud.	−42 −30 3	2.7	2.5	47
	24	L. IC	−9 −25 −11	2.7	2.5	101
	25	R. IC	18 −24 −8	3.8	3.2	1362
N0S0 - N0SR	26	R. Aud	41 −18 6	5.9*	4.4	1904
	27	R. Aud	50 −25 18	3.0	2.7	647
	28	L. Aud	−43 −17 7	3.1	2.7	229
NπS0 – N0Sπ	29	L. P. Thal.	−19 −21 14	3.0	2.7	59
N0Sπ – NπS0	30	L. Aud.	−33 −33 21	3.5	3.1	1009
	31	L. Aud	−52 −30 17	3.0	2.7	152
	32	CC	8 22 8	3.8	3.2	392
	33	R. MGB	27 −3 −7	2.9	2.6	308
N0S0 - NoStim	34	L. Aud.	−60 −15 32	6.6	4.7	38377
	35	R. Aud.	40 −29 9	6.8	4.8	20438
NoStim – N0S0	36	L,R IC	−8 −20 −8	5.3	4.1	4745
	37	Splenium	3 −20 25	5.2	4.1	3855
	38	CC	−1 34 11	4.4	3.6	1535
	39	L. P. Thal.	−23 −34 9	3.0	2.7	521
	40	Insula	27 24 −7	5.7	4.3	19421
N0Sπ - NoStim	41	L. Aud.	−36 −24 10	6.5	4.6	21245
	42	R. Aud.	63 −14 17	8.7	5.5	21631
NoStim – N0Sπ	43	IC	0 −26 −16	4.2	3.5	203 **
	44	Splenium	4 −30 22	3.4	3.0	364
N0Sπ - NoStim	45	L Aud.	−37 −26 8	5.7	4.3	34426
	46	R Aud.	44 −25 12	6.0	4.4	13447
NoStim – N0Sπ	47	CC	0 24 14	5.5	4.2	8111
N0SL - NoStim	48	L. Aud.	−36 −26 12	6.3	4.5	8264
	49	R. Aud.	39 −27 9	7.1	4.8	15601
NoStim – N0SL	50	CC	−6 27 11	6.5	4.6	2749
	51	Splenium	3 −30 24	5.4	4.1	2503
	52	IC	−11 −18 −4	5.1	4.0	246
N0SR - NoStim	53	L Aud.	−36 −32 13	6.3	4.5	20574
	54	R. Aud.	40 −26 12	5.5	4.1	6752
NoStim – N0SR	55	Splenium	−7 −35 20	6.2	4.5	13393
N0 - NoStim	56	L. Aud.	−44 −19 6	6.3	4.6	18853
	57	R. Aud.	48 −30 17	8.5	5.4	19281
NoStim – N0	58	IC	−5 −14 −13	3.0	2.7	54
	59	Splenium	−6 −18 29	5.2	4.1	5705
	60	CC	3 30 10	5.1	4.0	912 **
S0 – NoStim	61	L. Aud.	−44 −38 14	4.2	3.5	7006
	62	R. Aud.	34 −25 7	3.3	2.9	110
NoStim - S0	63	Splenium	−4 −18 31	5.0	3.9	3658
	64	IC	0 −34 −18	3.5	3.0	881
	65	CC	1 31 10	4.7	3.8	3366

fMRI SPM results for all experimental contrasts for hypothesized regions. L. and R. Aud. is used to identify the left and right (respectively) primary and secondary auditory regions identified by Rivier and Clarke using cellular staining. Other abbreviations we use for our regions of interests are Splenium (splenium of the corpus callosum), CC (body of the corpus callosum), IC (inferior Colliculus), P. Thal. (pulvinar thalamus), MGB (medial geniculate body). The size of the regions was calculated using a threshold of p<.01, except in cases where the region was especially large, and hard to localize, in which case we used the more stringent p<.001; these regions are marked with **. The table has three sections: 1) comparisons of dichotic conditions vs. N0S0 (rows 1–28), comparison of N0Sπ and NπS0 (rows 29–33), and the comparison of each sound condition with the NoStim condition. Section 1 regions that were significant after SVC are marked with *.

**Table 4:** DTI/Hearing Threshold Correlations.

Scan Condition	Row #	Diffusion Image	Correlation	Location	MNI Max	SpmT	SPM Z	Size
N0S0	1	FA	NEG	CC	−26 12 23	3.6	3.1	2111
	2	FA	POS	None				
	3	MD	NEG	Splenium, R. Pulv. Thal., R. Medial Geniculate Body	1 18 −4	4.4	3.6	8162
	4	MD	POS	None				
	5	AD	NEG	R. IC	10 −26 −17	3.4	3.0	115
	6	AD	NEG	Splenium,	17 −30 23	4.2	3.4	5893
				R. Pulv. Thal.				
	7	AD	POS	None				
N0Sπ	8	FA	NEG	LL	10 −19 −36	4.2	3.5	1590
	9	FA	POS	None				
	10	MD	NEG	None				
	11	MD	POS	None				
	12	AD	NEG	LL	3 −28 −40	3.2	2.8	316
	13	AD	POS	None				
NπS0	14	FA	NEG	LL	−8 −21 −38	4.0	3.4	963
	15	MD	NEG	LL	1 −22 −35	4.8	3.8	1912
	16	MD	POS	None				
	17	AD	NEG	LL	−2 −22 −36	5.2	4.0	2154
	18	AD	POS	None				
N0SL	19	FA	NEG	8n	27 −43 −39	4.5	3.7	898
	20	FA	NEG	CC	−16 11 22	2.8	2.5	16
	21	FA	POS	CC	18 22 20	3.6	3.1	92
	22	MD	NEG	LL/SOC	−2 −20 −32	3.1	2.8	132
	23	MD	POS	CC	−7 15 24	2.9	2.7	73
	24	AD	NEG	L. 8^th^ nerve, LL	−17 −34 −42	4.2	3.4	698
	25	AD	POS	CC	−5 15 25	3.7	2.9	180
N0SR	26	FA	NEG	CC	−25 23 5	3.6	3.1	75
	27	FA	NEG	CC neighbor	25 9 27	2.9	2.6	192
	28	FA	POS	IC	−10 −19 −7	4.3	3.5	1831
	29	FA	POS	CC	−15 −30 35	5.0	3.9	1046
	30	MD	NEG	LL	−11 −32 −44	5.1	4.0	2542
	31	MD	NEG	IC	−9 −16 −7	3.1	2.7	1082
	32	MD	NEG	L. Aud. Assoc.	−47 −20 1	3.2	2.8	1391
	33	MD	NEG	L. Aud. Assoc.	−49 −19 21	4.8	3.8	15176
	34	MD	POS	CC	−4 17 21	3.1	2.7	136
	35	AD	NEG	LL	−11 −32 −44	5.5	4.2	2381
	36	AD	NEG	L. Aud. Assoc.	−41 −14 25	4.9	3.8	15438
	37	AD	POS	CC	−2 15 22	3.3	2.9	849

DTI SPM results for all experimental correlation for hypothesized regions: splenium of the corpus callosum (splenium), body of the corpus callosum (CC), lateral lemniscus/superior olivary complex level of the brain stem (LL/SOC), pulvinar thalamus (Pulv. Thal.). Auditory Associative (Aud Assoc.) regions were not hypothesized because of the size of the region. 8^th^ nerve was not hypothesized because we anticipated very small alignment issues would hinder the findings.

### N0S0

The fMRI contrast N0S0 – NoStim had activation in the left and right auditory cortices (Table 3, rows 34 and 35), whereas the contrast, NoStim – N0S0, had a spatially large (4745 voxels) activation extending from the inferior colliculus to the red nucleus (Table 3, row 36). There was additional activation in the splenium of the corpus callosum, and left pulvinar thalamus (Table 3, rows 37–39). N0 – NoStim revealed activation in the left and right auditory cortex (Table 3, rows 56 and 57) with a slightly greater SPM t values than N0S0 – NoStim (Table 3, 34 and 35). Like NoStim – N0S0, both NoStim – N0 (Table 3, rows 58–60) and NoStim – S0 (Table 3, rows 63–65) also had activation in the inferior colliculus, splenium, and another region within the body of the corpus callosum. Hence either there was a deactivation with diotic conditions, or the activity of the inferior colliculus and regions of the corpus callosum is somewhat elevated for NoStim compared with the conditions N0S0, N0, and S0.

**Figure 1:**
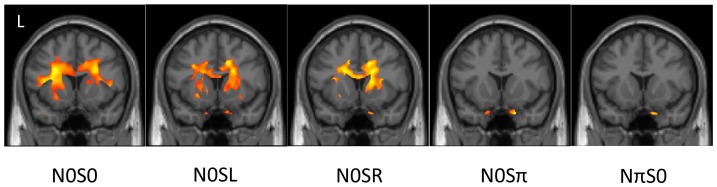
Regions of negative correlation between DTI FA measures and signal detection thresholds are shown for N0S0, N0SL, N0SR, N0Sπ, and NπS0. These show regions where tracts are more directional (anisotropic) with better thresholds. While there is a bilateral pattern revealed for N0S0, N0SL, and N0SR, there is no activation seen in this region for N0Sπ and NπS0. Each image is a coronal view, with the MNI template Y coordinate = 13 mm; this location was selected based on a finding of activity between N0Sπ and NπS0 in our previous study within the corpus callosum. A lenient threshold of p<.1, was used to show the potential full extent of the involved regions. Images are displayed with patient's left on the left side of the image, an “L” marks the patient's left on the first image.

Several regions showed negative correlations between signal detection thresholds for N0S0 and DTI measures of FA, MD, and AD. A region was found that was off center for the corpus callosum (SPM t max at MNI coordinate = −26, 12, 23 mm, Table 4, row 1) for our tested threshold (p<.01) for the FA images; but if we relax the significance level to p<.1, there is evidence of symmetrical bilateral network (Figure 1, left-most image). Negative correlations between N0S0 thresholds and MD and AD image values were found in the splenium of the corpus callosum and right pulvinar thalamus (Table 4, rows 3 and 6). Additionally there was a weak correlation between the AD image and N0S0 near the right inferior colliculus (Table 4, row 5). There were no significant positive correlations found between N0S0 thresholds and FA, MD, or AD values.

### N0SR and N0SL

**Figure 2:**
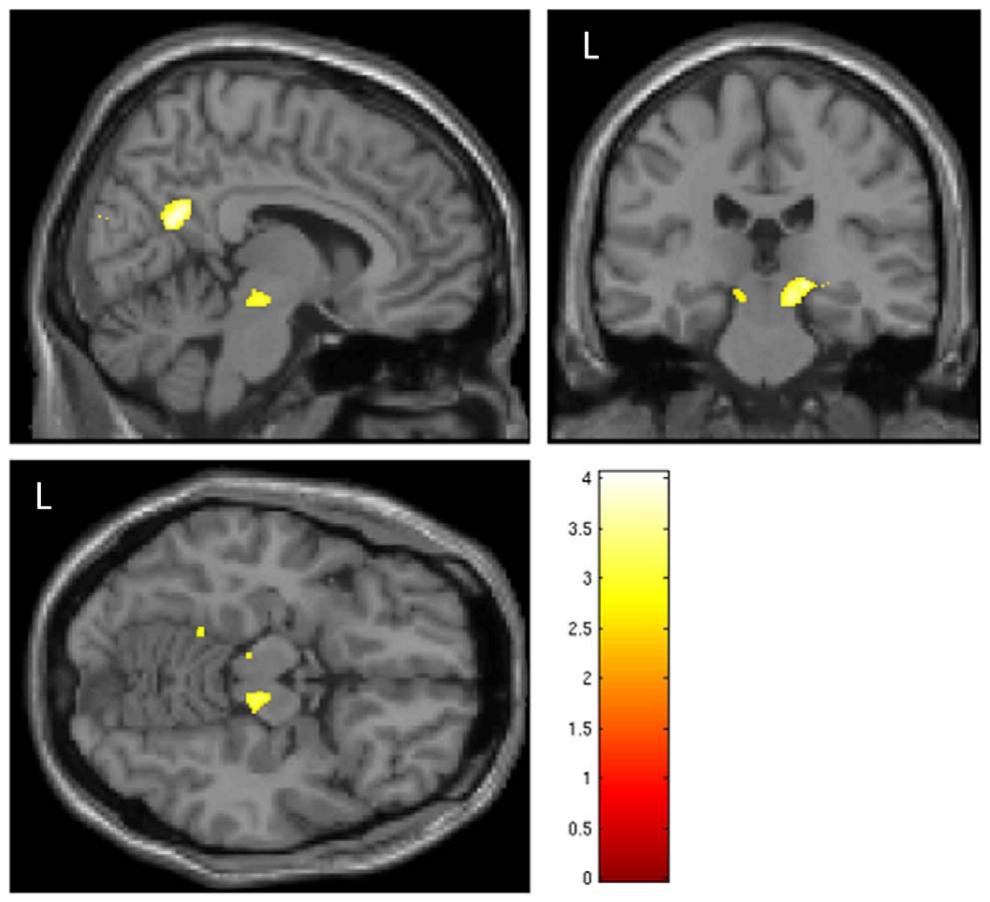
The fMRI contrast N0SR – N0S0 had activations within and near the Inferior Colliculus. A threshold of p<.01, uncorrected, was used to localize regions. Images are spatially normalized to the SPM MNI template, with orthogonal slices passing through the MNI coordinates 8, −26, −13 mm.

**Figure 3:**
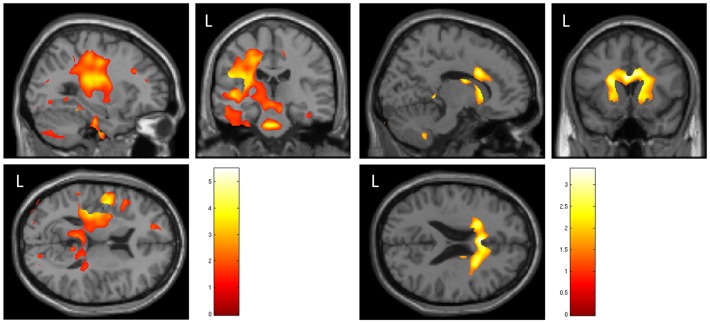
Left image: Negative correlation of N0SR detection level with AD measure of DTI. Right image: Positive correlation of N0SR detection level with AD measure of DTI. Images are threshold to give an exploratory view, at p<.1. None-the-less, there were several regions in the left hemisphere in the left image with SPM t values >4.0. Images are normalized to SPM MNI template, with orthogonal slices passing through the MNI coordinates: −29, −23,13 mm (left image, negative correlation) and MNI coordinates: 14, 15, 23 mm (right image, positive correlation).

For both fMRI contrasts, N0SL – NoStim and N0SR – NoStim, larger and stronger activations were observed in the left auditory cortex than the right auditory cortex (Table 3, rows 48 and 53 vs. rows 49 and 54). The contrast N0SR – N0S0 also had activation within and around the left and right inferior colliculus (Table 3, rows 24 and 25) that was more pronounced on the right (Figure 2). N0SL - N0S0 also had activation within the left pulvinar thalamus (Table 3, row 17). We also note evidence of activation within the left medial geniculate nucleus (Table 3, row 18), which we did not include in our hypothesis. The contrast N0S0 – N0SR had activation in the right auditory cortex, including the right planum polare (Table 3, row 26). Additionally, there was activation found in the left planum polare (Table 3, row 28). The contrast N0S0- N0SL had activation in the left auditory cortex and splenium of the corpus callosum (Table 3, rows 19–21). Correlations between MD image values and N0SR exhibited a patchy set of strong correlations, predominantly in the left hemisphere (Table 4, rows 32 and 33). When the threshold was relaxed to p<.1, an extensive region of left hemisphere activation was revealed, as shown in Figure 3. While this threshold level included many more voxels in the left hemisphere and revealed an activation pattern that encompassed the auditory pathway extending from the brainstem to auditory cortex, we did not find a correlation with any voxels leading to the right auditory cortex. In contrast, the pattern for the negative correlation of N0SL with MD only showed voxels within the brainstem that overlapped with the lateral lemniscus and superior olivary complex (Table 4, row 22).

### N0Sπ and NπS0

**Figure 4:**
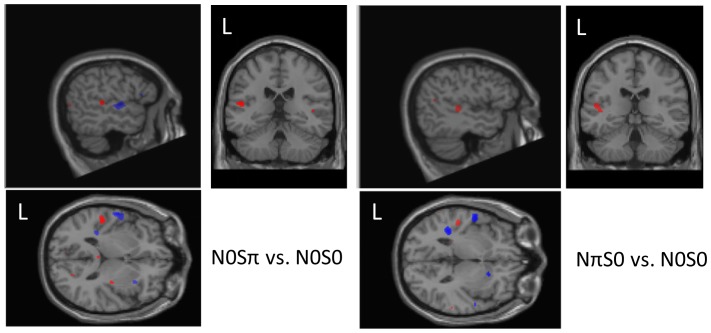
Red color indicates increased activity for dichotic condition versus N0S0. Blue indicates increased activity for N0S0 versus the dichotic condition. The overall patterns for N0Sπ vs. N0S0 (left image) and NπS0 vs. N0S0 (right image) are similar within the left auditory cortex. Regions are threshold at p<.01, uncorrected. Images are spatially normalized to SPM MNI template, but have been rotated (pitch) by .38 radians, to enable the view of the three regions of activation within the axial view. Orthogonal slices were selected to pass through the region of activation for N0Sπ – N0S0 (MNI coordinates: −52, −30, 1 mm) and NπS0 – N0S0 (MNI coordinates: −47, −27, −2 mm).

**Figure 5:**
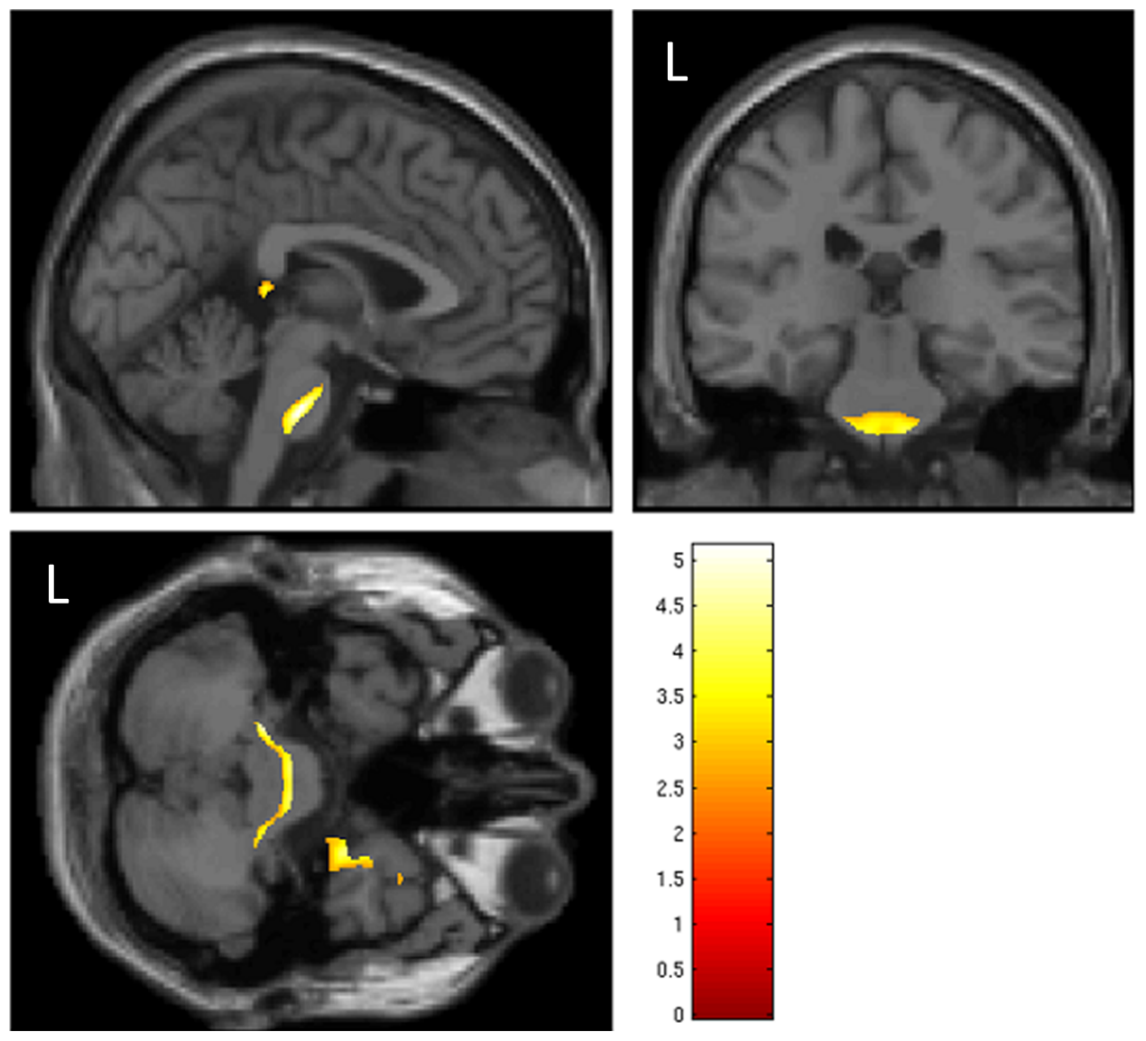
Negative correlation of AD measure of DTI with participants' NπS0 signal detection threshold. Activation threshold was set at p<.01, uncorrected, and is displayed on SPM MNI template. Displayed orthogonal slices pass through the MNI coordinates: −1, −27, −42 mm.

There is a strong similarity in the fMRI activation pattern for N0Sπ vs. N0S0 and NπS0 vs. N0S0. Both showed increased and decreased activation within auditory cortex regions (Table 3, Rows 1, 2, 5, 6, 8, 9, 11–14), with stronger activations seen in the left hemisphere (Figure 4). The contrast N0Sπ – N0S0 additionally showed a weak activation within the corpus callosum (Table 3, rows 3 and 4), and the contrast NπS0 – N0S0 additionally showed activation in the left pulvinar thalamus (Table 3, row 10). Negative correlations of signal detection threshold for both N0Sπ and NπS0, and FA, MD and AD DTI measures (Table 4, rows 8, 12, 14, 15, 17) were found in the lateral lemniscus (Figure 5). The contrast NπS0 – N0Sπ had a small activation in the left pulvinar thalamus (Table 3, row 29). The contrast N0Sπ – NπS0 had activation in the left auditory cortex and corpus callosum (Table 3, rows 30–32).

Post-hoc measures of the scanning room sound level of the fMRI sequence for the Toshiba scanner were 63 and 90 dB C, background and gradient sound level, respectively. The corresponding measures for the scanner used in our previous study were 61 and 97 dB C. Both sets are room measures made with a RadioShack Sound Level Meter, model 33-2055, facing the bore of the scanner, from a distance of 10 ft. from a phantom placed within the head holder. Measurements for our previously used scanner were made approximately 4 years after the collection of data, and sequences were reconstructed to duplicate what was originally used.

## Discussion

Not only were activations found in the auditory cortex for the dichotic condition (where the signal is perceived as louder) minus N0S0, but activations were also seen for the opposite contrasts, such as N0S0 – N0Sπ (Figure 4). These findings are in contrast to our previous study, where we predicted, but were unable to confirm neural correlates to MLD processing within the auditory cortex. The particular pattern of activation is of interest as the regions activated for N0S0 was postulated as “upstream” auditory association regions, whereas the region for N0Sπ and NπS0 was postulated as “downstream”, based on acetylcholinesterase staining [33]. While we had not hypothesized an efferent component to the MLD within the auditory cortex, we do not consider it unreasonable that the thalamus, inferior colliculi, or lower level structure could receive feedback from the auditory cortex during dichotic conditions. The novel aspect of the current study was the addition of DTI measures, which indicated several strong correlations between auditory regions of the DTI images and the participants' signal detection ability. DTI measures, FA, MD, and AD, give varying measure of the connectivity along fiber tracts. There was a strong negative correlation between detection thresholds for NπS0 and N0Sπ and voxels from the MD and AD images within the brainstem, especially at the level of the superior olivary complex, which indicates that higher MD and AD values were found in participants with better (lower) signal detection levels. Additionally we found spatially large regions, which extended from the brainstem to the left auditory cortex (Figure 3), with negative correlations between AD (and MD) and N0SR signal detection levels. This finding may help explain the phenomenon of the right ear advantage (REA) [36]. Overall, our DTI findings suggest that a subject's ability to detect a signal within noise may be dependent on the connectivity strength of the auditory fiber tracks. Finally, at a location in the corpus callosum where we found fMRI activation for NπS0 – N0Sπ in our previous study, we found a distinctive bilateral activation with DTI measures for some conditions (Figure 1), which we conjecture may be related to attention.

Our inclusion of DTI imaging allowed us to perform a correlational analysis between signal detection ability (within a noise background) and characteristics of the fiber tracts. To date, there have only been a few auditory studies that have incorporated DTI. For this reason we consider the DTI results to mainly exploratory, and we used lenient thresholds and multiple DTI measures. As binaural listening obviously requires a crossing of auditory information, our assumption was that these activation locations will reveal characteristics that correlate with the detection ability of the participants. As such our focus was on regions of the brainstem and the corpus callosum. That there should be a cortical component beyond a brainstem response to the MLD, we base on evoked potential studies [37]–[40], near-field evoked potential studies [5], and our previous study [10], [28]. Furthermore, our results also demonstrated differences in the processing path among the binaural conditions. Figure 5 shows a wide region extending bilaterally towards the 8^th^ nerve for the AD measure of DTI which correlated with NπS0 (Table 4, row 17). With the assumption that increased AD values imply better transfer of auditory information, this particular result may be an indication of the crucial need to preserve timing information when the majority of frequencies are out of phase. The region size for N0Sπ was much smaller than for NπS0.

Figure 3 may provide a partial clue into the phenomenon of REA. There is a very large volume of AD image voxels which correlated with signal detection levels in the left hemisphere, which extended from the inferior colliculus to the auditory cortex. While the weaker threshold reveals an extensive network of voxels in the left hemisphere, there was no indication of voxels leading to the right auditory cortex from the brainstem. In general, the negative correlation between DTI measures of fiber connectivity strength and signal detection threshold within a noise masker, suggests possible future applications for using DTI in investigating signals (including speech) in noise. There has only been a limited use of DTI for auditory studies but these include the investigation of tinnitus [41], [42], language laterality in autism [43], sensorineural hearing loss [44], and auditory processing disorder [27].

Rivier and Clarke (Rivier and Clarke, 1997) performed staining (cytochrome oxidase, acetylcholinesterase and NADPH-diaphorase) of the supratemporal and insular cortex of human brains. Based on their findings of staining patterns, they proposed several distinct regions, including Anterior Area (AA), Lateral Area (LA), and Posterior Area (PA). A diagram of the individual locations is provided in Figure 10 of their work, and is redrawn together with maps from others in Figure 7 of Talavage et al. (2004). Based on the number and layer distribution of pyramidal neurons and axons, Rivier and Clarke conjectured that AA and PA regions were “upstream” regions, whereas LA was “downstream”. We believe the regions given for row 1 and row 8, Table 3, correspond to the region LA, for contrasts N0Sπ – N0S0 and NπS0 – N0S0, respectively. While rows 5, 6, 11, 12 correspond to the AA and PA regions, for the opposite contrasts. All regions are visible in Figure 4. While auditory neural processing is generally modeled as a chain of afferent connections, the efferent pathways connecting the auditory cortex to cochlea have been investigated in chinchillas [45]. Furthermore, Khalfa et al. studied epilepsy patients pre and post superior temporal gyrus excision [46] and found an effect on the peripheral auditory system. Finally, there has also been recent work investigating [47] and modeling [48] the medial olivocochlear (MOC) auditory efferent system located in the brainstem to improve the detection and intelligibility of speech in noise.

By using SVC rather than family-wise error (FWE) correction, and enrolling 20 subjects, we were able to detect neural correlates within the auditory cortex for MLD contrasts. While not all of our reported regions reach SVC significance, 6 of 10 regions reported within the left auditory cortex did. That the activations were stronger in the left hemisphere is consistent with the findings of Stracke et al. who, using MEG, found overall dominance of the left hemisphere for signal in noise processing [49]. The increased activity for N0S0 over the dichotic conditions (N0Sπ and NπS0) is in seeming conflict with the findings of Ernst et al. [50], who found correlation between regional activity level within the auditory cortex and stimuli signal level, in similar locations as our AA and PA. However, unlike Ernst's study, the true signal level in our study remains the same. That we found an actual decrease could indicate a different processing strategy between the dichotic and diotic conditions within AA and PP regions (Table 3, Rows 5, 6, 11, 12; Figure 4). Consistent with our current findings, we previously found, using positron emission tomography, higher activity for the lowest intensity signals (presented without noise), than for slightly more moderate levels [51]. Our interpretation of the current results is that there is an extra processing effort to hear the (near threshold) signal for N0S0.

In our previous study we identified a region of the corpus callosum (MNI coordinate = 10, 13, 16 mm) that appeared to be involved with the processing of MLD, located within the body of the corpus callosum. This region of the corpus callosum is difficult to explain with our data. Previously we found activation for NπS0 – N0Sπ at this location; however, within 1 cm in our current study we found activation for N0Sπ – NπS0 (Table 3, row 32). In Figure 1, we show coronal images through the voxel with the maximum SPM t value from our previous study within the corpus callosum (MNI coordinate = 10 13 16 mm) for correlations for each condition using FA. This figure shows a weak but extensive bilateral negative correlation between FA values and thresholds for N0S0, N0SL, and N0SR, but not N0Sπ and NπS0. Furthermore, N0SR shows an opposite correlation when MD is used instead of FA (compare Figures 1 and 3). While this region appears to be playing a role with binaural listening, we are left with many questions. Our conjecture is that this region relates to the “strategy” that a listener may take to listen for the tone. The activated regions extend bilaterally into the lateral and medial caudate, accumbens nucleus, and putamen. These regions are known to be associated with reward and attention [52], and may be further regulated by dopamine release [53]. As noted in our previous study, there was a significant resonance in response to the noise portion of the stimuli that was well outside of the desired 400–600 Hz band-passed noise portion of the stimuli. While we demonstrated that our previous subjects had an MLD using the scanner headphones, it was reduced from the soundbooth measures. Hence, the broader spectrum of presented noise in our previous study may have led to a different executive level strategy or “concentration” level than in this study for the different conditions.

In our previous paper, we argued that our results were consistent with the Kimura model [6], and that the pulvinar thalamus acts as a gating mechanism for binaural listening. This was supported (in the previous study) by findings of activation the pulvinar thalamus, corpus callosum and insulae for the contrast NπS0 – N0Sπ. However, in that experiment we found little support from our direct comparison contrasts: NπS0 – N0S0 and N0Sπ – N0S0. In this study we found evidence of the involvement of the pulvinar thalamus, although it was weaker than previously and located in the left and not right hemisphere. We did not include the insulae as a region of interest in this study; however, we did not find the wide spread insular involvement for the contrast NπS0 – NπS0 as was seen previously. With an increased number of subjects and non-FWE corrected statistics, we hypothesized that correlates to the MLD could be detected within the auditory cortex and the inferior colliculus. Since we specified our regions of interest, we chose a voxel-wise threshold for reporting activations using p<.01, uncorrected. We also provided the maximum value of SPM t and SPM Z for each region, and marked regions with an * that were significant after SVC for multiple comparisons, to give the reader a better indication of significance of the found region.

While our study was designed to duplicate our previous conditions to allow us to further investigate our original findings, it was performed at a new location using a different vendor's scanner (Toshiba Titan 3T scanner), which was 7 dB quieter [54]. We believe that the decreased scanner noise may have helped to improve the overall comfort level of the subjects. This is supported by the reduced mean number of scan volumes per subject that were identified as outliers in the current study (2.9 vs. 16.1 previously), since a common cause of outliers is small motion by a participant who may not be at ease within the scanner. Furthermore, regions of activation within the auditory cortex for fMRI noise/signal contrasts that were compared to no-stimuli condition had improved activation overall in our current study.

## Conclusions

This study revealed a neural correlate to the MLD within the auditory cortex that we were unable to obtain in our previous study. We also provided fMRI evidence for brainstem and corpus callosum involvement in the neural processing of the MLD. Furthermore, our addition of DTI measures show that all AD, MD, and FA DTI measures are valuable in further understanding MLDs. Our results show a strong correlation between regions of the brainstem and cortical regions with signal detection thresholds used in the MLD.

## References

[1] Hirsh IJ (1948) The influence of interaural phase on interaural summation and inhibition. The Journal of the Acoustical Society of America 20: 536–544.

[2] Licklider JCR (1948) The influence of interaural phase relations upon the masking of speech by white noise. The Journal of the Acoustical Society of America 20: 150–159.

[3] Jiang D, McAlpine D, Palmer AR (1997) Responses of neurons in the inferior colliculus to binaural masking level difference stimuli measured by rate-versus-level functions. Journal of Neurophysiology 77: 3085–3106. 9212259 10.1152/jn.1997.77.6.3085

[4] Palmer AR, Jiang D, McAlpine D (2000) Neural responses in the inferior colliculus to binaural masking level differences created by inverting the noise in one ear. Journal of Neurophysiology 84: 844–852. 10938311 10.1152/jn.2000.84.2.844

[5] Guo Y, Burkard R (2003) The masking level difference in chinchilla auditory cortex. Effects of inner hair cell loss. Hearing Research 178: 12–26. 12684173 10.1016/s0378-5955(03)00023-6

[6] Kimura D (1967) Functional asymmetry of the brain in dichotic listening. Cortex 3: 163–178.

[7] Sugishita M, Otomo K, Yamazaki K, Shimizu H, Yoshioka M, et al (1995) Dichotic listening in patients with partial section of the corpus callosum. Brain 118: 417–427. 7735883 10.1093/brain/118.2.417

[8] Ojemann G (1975) Language and the thalamus: object naming and recall during and after thalamic stimulation. Brain and Language 2: 101–120. 1100194 10.1016/s0093-934x(75)80057-5

[9] Hugdahl K, Wester K, Asbjornsen A (1990) The role of the left and right thalamus in language asymmetry: Dichotic listening in Parkinson patients undergoing stereotactic thalamotomy. Brain and Language 39: 1–13. 2207615 10.1016/0093-934x(90)90001-w

[10] Wack DS, Cox JL, Schirda CV, Magnano CR, Sussman JE, et al (2012) Functional anatomy of the masking level difference, an fMRI study. PloS one 7: e41263. 22848453 10.1371/journal.pone.0041263 PMC3407245

[11] Karbe H, Herholz K, Halber M, Heiss W (1998) Collateral inhibition of transcallosal activity facilitates functional brain asymmetry. Journal of Cerebral Blood Flow & Metabolism 18: 1157–1161. 9778192 10.1097/00004647-199810000-00012

[12] Sokoloff L (1977) Relation between physiological function and energy metabolism in the central nervous system. J Neurochem 29: 13–26. 407330 10.1111/j.1471-4159.1977.tb03919.x

[13] Tettamanti M, Paulesu E, Scifo P, Maravita A, Fazio F, et al (2002) Interhemispheric transmission of visuomotor information in humans: fMRI evidence. Journal of Neurophysiology 88: 1051–1058. 12163553 10.1152/jn.2002.88.2.1051

[14] Fabri M, Polonara G (2013) Functional Topography of Human Corpus Callosum: An fMRI Mapping Study. Neural Plasticity 2013 10.1155/2013/251308 PMC3586479 23476810

[15] Buxton RB (2009) Introduction to functional magnetic resonance imaging: principles and techniques: Cambridge University Press.

[16] Huettel SA, Song AW, McCarthy G (2004) Functional magnetic resonance imaging: Sinauer Associates Sunderland.

[17] Friston KJ, Ashburner JT, Kiebel SJ, Nichols TE, Penny WD (2011) Statistical Parametric Mapping: The Analysis of Functional Brain Images: The Analysis of Functional Brain Images: Academic Press.

[18] Budd TW, Hall DA, Gonçalves MS, Akeroyd MA, Foster JR, et al (2003) Binaural specialisation in human auditory cortex: an fMRI investigation of interaural correlation sensitivity. Neuroimage 20: 1783–1794. 14642488 10.1016/j.neuroimage.2003.07.026

[19] Ernst S, Uppenkamp S, Verhey JL (2010) Cortical representation of release from auditory masking. Neuroimage 49: 835–842. 19616635 10.1016/j.neuroimage.2009.07.014

[20] Hall DA, Plack CJ (2007) The human “pitch center” responds differently to iterated noise and Huggins pitch. Neuroreport 18: 323–327. 17435596 10.1097/WNR.0b013e32802b70ce

[21] Hall DA, Plack CJ (2009) Pitch processing sites in the human auditory brain. Cerebral Cortex 19: 576–585. 18603609 10.1093/cercor/bhn108 PMC2638814

[22] Chait M, Poeppel D, Simon JZ (2006) Neural response correlates of detection of monaurally and binaurally created pitches in humans. Cerebral Cortex 16: 835–848. 16151180 10.1093/cercor/bhj027

[23] Barker D, Plack CJ, Hall DA (2011) Reexamining the evidence for a pitch-sensitive region: A human fMRI study using iterated ripple noise. Cerebral Cortex 22: 745–763. 21709174 10.1093/cercor/bhr065

[24] Westerhausen R, Grüner R, Specht K, Hugdahl K (2009) Functional relevance of interindividual differences in temporal lobe callosal pathways: a DTI tractography study. Cerebral Cortex 19: 1322–1329. 18842665 10.1093/cercor/bhn173

[25] Musiek FE, Weihing J (2011) Perspectives on dichotic listening and the corpus callosum. Brain and cognition 76: 225–232. 21531063 10.1016/j.bandc.2011.03.011

[26] Seok J-H, Park H-J, Chun J-W, Lee S-K, Cho HS, et al (2007) White matter abnormalities associated with auditory hallucinations in schizophrenia: a combined study of voxel-based analyses of diffusion tensor imaging and structural magnetic resonance imaging. Psychiatry research 156: 93. 17884391 10.1016/j.pscychresns.2007.02.002

[27] Schmithorst VJ, Farah R, Keith RW (2013) Left ear advantage in speech-related dichotic listening is not specific to auditory processing disorder in children: A machine-learning fMRI and DTI study. NeuroImage: Clinical 10.1016/j.nicl.2013.06.016 PMC3791276 24179844

[28] Wack DS (2010) Localization of the binaural mechanisms underlying masking level differences, using fMRI: State University of New York at Buffalo.

[29] Hall DA, Haggard MP, Akeroyd MA, Palmer AR, Summerfield AQ, et al (1999) “Sparse” temporal sampling in auditory fMRI. Human Brain Mapping 7: 213–223. 10194620 10.1002/(SICI)1097-0193(1999)7:3<213::AID-HBM5>3.0.CO;2-N PMC6873323

[30] Edmister WB, Talavage TM, Ledden PJ, Weisskoff RM (1999) Improved auditory cortex imaging using clustered volume acquisitions. Human Brain Mapping 7: 89–97. 9950066 10.1002/(SICI)1097-0193(1999)7:2<89::AID-HBM2>3.0.CO;2-N PMC6873308

[31] Luo W, Nichols T (2003) Diagnosis and exploration of massively univariate neuroimaging models. Neuroimage 19: 1014–1032. 12880829 10.1016/s1053-8119(03)00149-6

[32] Ashburner J, Friston K (1997) The role of registration and spatial normalisation in detecting activations in functional imaging. CLINICAL MRI 7: 26–27.

[33] Rivier F, Clarke S (1997) Cytochrome oxidase, acetylcholinesterase, and NADPH-diaphorase staining in human supratemporal and insular cortex: evidence for multiple auditory areas. Neuroimage 6: 288–304. 9417972 10.1006/nimg.1997.0304

[34] Mai JK, Assheuer J, Paxinos G (1997) Atlas of the human brain: Academic Press San Diego:.

[35] Behrens T, Woolrich M, Jenkinson M, Johansen-Berg H, Nunes R, et al (2003) Characterization and propagation of uncertainty in diffusion-weighted MR imaging. Magnetic Resonance in Medicine 50: 1077–1088. 14587019 10.1002/mrm.10609

[36] Shankweiler D, Studdert-Kennedy M (1967) Identification of consonants and vowels presented to left and right ears. The Quarterly journal of experimental psychology 19: 59–63. 6041684 10.1080/14640746708400069

[37] Fowler CG, Mikami CM (1992) The late auditory evoked potential masking-level difference as a function of noise level. Journal of Speech, Language and Hearing Research 35: 216–221. 10.1044/jshr.3501.216 1735971

[38] Fowler CG, Mikami CM (1995) Binaural phase effects in the auditory brainstem response. J Am Acad Audiol 6: 399–406. 8580499

[39] Fowler CG, Mikami CM (1996) Phase effects on the middle and late auditory evoked potentials. J Am Acad Audiol 7: 23–30. 8718489

[40] Wong WYS, Stapells DR (2004) Brain stem and cortical mechanisms underlying the binaural masking level difference in humans: An auditory steady-state response study. Ear & Hearing 25: 57–67. 14770018 10.1097/01.AUD.0000111257.11898.64

[41] Husain FT, Medina RE, Davis CW, Szymko-Bennett Y, Simonyan K, et al (2011) Neuroanatomical changes due to hearing loss and chronic tinnitus: a combined VBM and DTI study. Brain research 1369: 74–88. 21047501 10.1016/j.brainres.2010.10.095 PMC3018274

[42] Crippa A, Lanting CP, Van Dijk P, Roerdink JB (2010) A diffusion tensor imaging study on the auditory system and tinnitus. The open neuroimaging journal 4: 16–25. 20922048 10.2174/1874440001004010016 PMC2948149

[43] Knaus TA, Silver AM, Kennedy M, Lindgren KA, Dominick KC, et al (2010) Language laterality in autism spectrum disorder and typical controls: a functional, volumetric, and diffusion tensor MRI study. Brain and language 112: 113–120. 20031197 10.1016/j.bandl.2009.11.005 PMC2822339

[44] Lin Y, Wang J, Wu C, Wai Y, Yu J, et al (2008) Diffusion tensor imaging of the auditory pathway in sensorineural hearing loss: changes in radial diffusivity and diffusion anisotropy. Journal of Magnetic Resonance Imaging 28: 598–603. 18777540 10.1002/jmri.21464

[45] León A, Elgueda D, Silva MA, Hamamé CM, Delano PH (2012) Auditory Cortex Basal Activity Modulates Cochlear Responses in Chinchillas. PloS one 7: e36203. 22558383 10.1371/journal.pone.0036203 PMC3340362

[46] Khalfa S, Bougeard R, Morand N, Veuillet E, Isnard J, et al (2001) Evidence of peripheral auditory activity modulation by the auditory cortex in humans. Neuroscience 104: 347–358. 11377839 10.1016/s0306-4522(01)00072-0

[47] Kim S, Frisina RD, Frisina DR (2006) Effects of age on speech understanding in normal hearing listeners: Relationship between the auditory efferent system and speech intelligibility in noise. Speech communication 48: 855–862.

[48] Smalt CJ (2012) Functional magnetic resonance imaging (fMRI) of a real-time cochlear implant acoustic simulation and auditory modeling of the medial olivocochlear efferent system.

[49] Stracke H, Okamoto H, Pantev C (2009) Interhemispheric support during demanding auditory signal-in-noise processing. Cerebral Cortex 19: 1440–1447. 18936273 10.1093/cercor/bhn183

[50] Ernst S, Verhey JL, Uppenkamp S (2008) Spatial dissociation of changes of level and signal-to-noise ratio in auditory cortex for tones in noise. Neuroimage 43: 321–328. 18722535 10.1016/j.neuroimage.2008.07.046

[51] Lockwood AH, Salvi RJ, Coad ML, Arnold SA, Wack DS, et al (1999) The functional anatomy of the normal human auditory system: responses to 0.5 and 4.0 kHz tones at varied intensities. Cerebral Cortex 9: 65–76. 10022496 10.1093/cercor/9.1.65

[52] Haruno M, Kawato M (2006) Different neural correlates of reward expectation and reward expectation error in the putamen and caudate nucleus during stimulus-action-reward association learning. Journal of neurophysiology 95: 948–959. 16192338 10.1152/jn.00382.2005

[53] Badgaiyan RD, Wack D (2011) Evidence of dopaminergic processing of executive inhibition. PloS one 6: e28075. 22162756 10.1371/journal.pone.0028075 PMC3230601

[54] Katsunuma A, Takamori H, Sakakura Y, Hamamura Y, Ogo Y, et al (2001) Quiet MRI with novel acoustic noise reduction. Magnetic Resonance Materials in Physics, Biology and Medicine 13: 139–144. 10.1007/BF02678588 11755088

